# Antibiotic drug discovery

**DOI:** 10.1111/1751-7915.12388

**Published:** 2016-07-29

**Authors:** Wolfgang Wohlleben, Yvonne Mast, Evi Stegmann, Nadine Ziemert

**Affiliations:** ^1^ Interfaculty Institute of Microbiology and Infection Medicine TuebingenMicrobiology/BiotechnologyUniversity of TuebingenAuf der Morgenstelle 2872076TuebingenGermany; ^2^ German Center for Infection Research (DZIF)Partner Site TuebingenTuebingenGermany

## Abstract

Due to the threat posed by the increase of highly resistant pathogenic bacteria, there is an urgent need for new antibiotics; all the more so since in the last 20 years, the approval for new antibacterial agents had decreased. The field of natural product discovery has undergone a tremendous development over the past few years. This has been the consequence of several new and revolutionizing drug discovery and development techniques, which is initiating a ‘New Age of Antibiotic Discovery’. In this review, we concentrate on the most significant discovery approaches during the last and present years and comment on the challenges facing the community in the coming years.

## Introduction

Nature has been a source of medicinal products for millennia, with many useful active substances developed from plant sources. In the 20th century, the discovery of the penicillins was the starting point for drug discovery from microbial sources. The majority of drugs have been developed from lead structures on the basis of natural products synthesized by bacteria. Drugs derived from bacterial secondary metabolites are in manifold use, for example in diagnosis, mitigation, or in the treatment, or prevention of a disease or relief of discomfort.

It is estimated that during the golden era (1940–1970) of microbial natural product screening, tens of millions of soil microorganisms have been screened (Baltz, 2005), an enormous effort that provided the vast majority of microbial metabolites known today (Bérdy, 2005, 2012; Monciardini *et al*., 2014). These substances include widely prescribed antibacterial therapeutics, such as erythromycin, streptomycin, tetracycline, vancomycin and chemotherapeutic drugs such as doxorubicin. Ninety per cent of all antibiotics used in clinics today are derived from microorganisms (Bérdy, 2005; Katz and Baltz, 2016). Presently, more than 23 000 natural products (Katz and Baltz, 2016) with antibacterial activity are known, which are produced from microorganisms, compared to only 25 000 isolated from higher organisms including plants and animals. For a compilation of the most important compounds, see Katz and Baltz (2016). Out of this high number of compounds, only about hundreds are used in clinical practice (Spížek *et al*., 2016). Although the numbers are only roughly estimated, among eubacteria actinobacteria seem to be the most efficient antibiotic producers.

Three different approaches have been applied for effective drug discovery programmes:

1. Historically, substances, crude extracts or purified chemicals were screened for biological activity mostly in whole cell‐assays without knowing the drug target. Only after an active substance has been identified, serious efforts have been made to analyse the target and the mode of action of the compound. This strategy is known as *bioactive‐guided screening*, classical pharmacology, forward pharmacology (Takenaka, 2001) or phenotypic drug discovery (Lee *et al*., 2012).
2. Another approach to identify new drug substances is denominated as *chemical screening*, which aims to identify novel, chemically diverse molecules without taking into account their biological activity. The substances used in this approach can originate from biological sources (such as metabolites from microorganisms) or from chemical libraries. For this, sophisticated analytical methods are applied, such as high‐performance liquid chromatography, mass spectrometry (MS) or nuclear magnetic resonance spectroscopy. Hereby, the structure elucidation is a pivotal/crucial step to avoid the re‐discovery of an already known substance. Nowadays, large databases of mass spectra for known compounds are available and can efficiently be used for dereplication. If, thus, a new substance has been identified, it is used in assays to search for biological (antibiotic) activity.
3. In contrast to the chemical screening, the *target‐oriented screening* aims to identify compounds that hit a known and validated molecular target. Thereby, the target represents a cellular or molecular structure involved in the pathology of interest that the drug‐in‐development is meant to act on. Different attributes of the target have to be taken into account. For example, a good antibacterial target has no human homologue and is present in a wide range of bacteria where it is essential. Further important attributes are the location of the target in bacteria and a low frequency of resistance to new compounds. Hence, multiple targets or targets encoded by multiple genes should be selected because high‐level, target‐based resistance to these compounds does not occur by single‐step mutations.

The main disadvantage of these screening programmes was that numerous metabolites have been repeatedly rediscovered (Genilloud *et al*., 2011). This has led to estimates that the rate of discovery of different classes of microbial metabolites can vary significantly in ‘random’ screening campaigns (Baltz, 2007). Due to this empirical knowledge, it is assumed that most of the low‐hanging fruits have already been picked and only a few new metabolites are left to be harvested unless massive screening programmes are implemented (Zengler *et al*., 2002; Baltz, 2007, Monciardini *et al*., 2014).

To overcome this problem, *combinatorial chemistry* has been developed as a key technology enabling the generation of large screening libraries for the needs of high‐throughput screenings. However, now, after more than two decades of combinatorial chemistry, it became apparent that despite the increased number of new chemical substances, no increase in lead structures or drug candidates has been reached (Newman and Cragg, 2007). Instead, the synthetic, combinatorial library compounds seem to cover only a limited and quite uniform chemical space (Rosén *et al*., 2009), whereas existing drugs and particularly natural products exhibit much greater chemical diversity (Feher and Schmidt, 2003).

## General principles of antibiotic biosynthesis

The actinobacterial secondary metabolome is a source for many compounds. Secondary metabolites are specialized compounds that are distinguished from primary metabolites because they are not directly involved in cell growth or reproduction. They usually are taxonomically restricted and have specialized functions, including signalling, nutrient acquisition or defence (Demain and Fang, 2000; Davies, 2013). The diversity of secondary metabolites is enormous, although their biosynthesis in principle is based on variations of only a few predominant pathways, viz. synthesis of polyketides (PKS), non‐ribosomal peptides (NRPS) or polypeptides, terpenes or sterols, etc., or combinations thereof. The specificity of individual biosynthetic enzymes determines which building blocks will be incorporated into the compound and how it will be modified. The knowledge on enzyme specificity can be used to predict the resultant compounds (Weber *et al*., 2015).

The enzymes catalysing secondary metabolic biosynthesis are encoded by genes, which generally are organized in so‐called biosynthetic gene clusters. Such a biosynthetic gene cluster is defined as the genome region, which harbours all genes required for the synthesis of the natural product as well as genes for the resistance, export and pathway‐specific regulation.

## Novel approaches for drug discovery

### Drug discovery using unexplored strains

Although *Actinobacteria* are a treasure chest for novel natural compounds, presumably only less than 1% of actinobacterial species have been cultivated so far meaning that around 99% of the population is unexplored (Davies, 1999). One main obstacle in the identification of novel species is that many bacteria cannot be cultured under conventional laboratory conditions. However, in recent years, several new and revolutionary cultivation techniques have been developed, which now allow to grow a broad range of so far uncultivable bacteria. One of these new methods is represented by the microfluidic bioreactor cultivation, which provides a high‐throughput cultivation system where up to 600 000 pure soil‐derived *Actinobacteria* can be grown in microfluidic droplets per hour (Zang *et al*., 2013). This technique allows to cultivate slow growers that under normal conditions would be outcompeted by fast‐growing strains but potentially may be promising natural compound producers. Actually, the idea of focusing on slow‐growing and/or hard‐to‐isolate strains has been one basis to set up the company Naicons in 2006, which aims to establish a large library of so far uncultured or unclassified actinomycetes and filamentous fungi and to isolate new metabolites thereof. This screening approach already led to the discovery of several new effective natural compounds, such as the protein synthesis inhibitor orthoformimycin, which is a structurally novel substance that follows a completely new mechanism of action or the class III lanthipeptide NAI‐112, which is a potent anti‐inflammatory agent (Monciardini *et al*., 2014). Another recent successful example for the identification of a novel anti‐infective substance from a previously uncultured bacterium is represented by teixobactin, which is a substance from the novel species of β‐proteobacteria named *Eleftheria terrae* (Ling *et al*., 2015). *E. terrae* was isolated with the help of a new cultivation technique, the so‐called iChip, which is a multichannel device composed of several hundred miniature diffusion chambers, each inoculated with a single environmental cell. The iChip then is placed back in the natural environment, which leads to a significantly increased colony count that subsequently can efficiently be cultivated under laboratory conditions (Nichols *et al*., 2010).

Besides sophisticated cultivation methods, also cutting‐edge methodologies, such as Next‐Generation Sequencing techniques, can be applied as efficient tool for the identification of novel natural compounds. An intriguing example is represented by the single‐cell and metagenomics‐based approach leading to the identification of the novel bacterial taxon ‘*Entotheonella*’ that co‐inhabits the Red sea marine sponge *Theonella swinhoei*. Metagenomic analysis showed that more than 40 bioactive polyketides and modified peptides belonging to seven different structural classes are produced by the endosymbiont in association with the sponge (Wilson *et al*., 2014).

Even, rather ‘classic’ approaches to search for novel antibiotics can still be very successful if samples are taken from unexplored habitats (e.g. desert, deep sea, endosymbiotic environment) or bacteria are investigated, which do not belong to the classes of well‐known antibiotic producers.

A successful example is the relatively novel genus *Salinispora*, of which several species have been isolated from marine sediments of Guam, Palau and the Red Sea producing salinosporamides, inhibitors of the 20S proteasome (Jensen *et al*., 2015). A promising underexplored habitat is also the human body: Recently, it has been shown that the nasal colonizing organism *Staphylococcus lugduensis* produces *a* novel peptide antibiotic (Peschel pers commun; *Nature* in press). This shows that also non‐actinomycetal bacteria can be promising sources for novel antimicrobials. This is also exemplified by the production of several new antibiotics by anaerobiers, such as *Clostridium beijerinckii*, which recently has been shown to produce the pentacyclic polyphenol clostrubin, which is a highly active substance against diverse pathogenic bacteria (Letzel *et al*., 2013; Pidot *et al*., 2014; Shabuer *et al*., 2015).

### Genome mining

In contrast to screening for chemical compounds, an alternative strategy has become increasingly popular during the last two decades (Ziemert *et al*., 2016). The so‐called genome mining approach detects and analyses the biosynthetic gene clusters of the chemical compounds and subsequently connects those genes to molecules. Genome mining has various advantages. The vast amount of DNA data available these days provides a large pool of potential compounds encoded in these genomes that, given the bioinformatics tools that exist, are relatively fast and easy to screen for almost no costs. Moreover, sophisticated web‐based tools, such as anti‐SMASH (Medema *et al*., 2011; Blin *et al*., 2013, 2014; Weber *et al*., 2015), PRISM (Skinnider *et al*., 2015) or NaPDoS (Ziemert *et al*., 2012), are easily accessible and do not require extended expertise in natural product biosynthesis or bioinformatics. Mining bacteria for their genetic potential also revealed that many more bacteria have the ability to produce natural products than previously thought and that more chemical diversity is waiting for discovery.

Based on structure and content of the biosynthetic gene clusters, chemical classes and structures of the encoded compound can be predicted. This information can be used to either guide a more targeted drug discovery technique (such as reactivity‐guided isolation; Castro‐Falcón *et al*., 2016) or peptide and glycogenomic approaches (Kersten *et al*., 2011, 2013) or allow the heterologous expression in an optimized expression host (*activation of silent gene cluster*). Furthermore, comprehensive biosynthetic gene cluster databases such as MiBIG (Minimum Information about a Biosynthetic Gene cluster) provide the possibility to easily estimate novelty in the encoded compounds (Medema *et al*., 2015).

However, it still remains challenging to connect gene clusters to bioactivity. Purely by predicting structures, it is often impossible to say if the encoded compounds have antibiotic or medically relevant activities and are worth further investigations. A recently developed method, therefore, combines the search for biosynthesis genes with detecting potential resistance genes. This so‐called target‐directed genome mining (Tang *et al*., 2015) is based on the observation that antibiotic‐producing bacteria need to be resistant against the producing compound themselves to avoid suicide. In some cases, a resistant second copy of the target gene is encoded within the biosynthetic gene cluster of the antibiotic allowing a more targeted search for antibiotic compounds with interesting targets.

A lot of the genome mining methods are based on the detection of domains and protein families well known to be involved in secondary metabolism such as PKS and NRPS. To overcome possible limitations and provide the opportunities to find biosynthetically unique chemistry and enzymes, two methods have been recently developed. The ClusterFinder algorithm by Cimermancic *et al*. (2014) is based on a probabilistic approach that compares the presence of Pfam domains of cluster involved in secondary metabolism compared with random areas in the genome. As proof of principles, the authors screened more than 1000 bacterial genomes and detected about 30 000 putative biosynthetic gene clusters. Another method that addresses the problem of finding novel pathways is based on evolutionary distances and was called EvoMining (Cruz‐Morales *et al*., 2016). EvoMining is based on the assumption that most enzymes used in secondary metabolism have been duplicated and evolved from primary metabolism (Cruz‐Morales *et al*., 2016). By recapitulating the evolutionary history of 23 enzyme families previously uninvestigated in the context of natural product biosynthesis, biosynthetic gene clusters coding for hidden chemical diversity were discovered. This phylogenomic analysis resulted in the discovery of arseno‐organic metabolites in *Streptomycetes coelicolor* and *Streptomyces lividans* (Cruz‐Morales *et al*., 2016).

A lot of efforts are focusing now on the implementation of the variety of genome mining methods and computational predictions into analytical chemistry and molecular biology approaches to allow a more streamlined drug discovery process. Methods such as peptide and glycogenomics (Kersten *et al*., 2011, 2013) connect computational predictions with state of the art mass spectrometric methods (*alternative methods for drug discovery*), whereas pattern‐based genome mining, for example, uses the huge amount of DNA sequence data available in a more comparative genomic approach combined with MS analysis (Duncan *et al*., 2015).

### Activation of silent gene cluster

Genome mining tools uncovered a plethora of so‐called silent gene clusters in diverse actinobacterial genomes. These clusters encode the synthesis of potential secondary metabolites that are not produced under standard laboratory culture conditions. In the past few years, a lot of inventive effort has been put into strategies to activate such silent gene clusters in order to find new secondary metabolites. For this, different expression strains have been developed to facilitate gene cluster expression and metabolite detection. For instance, the model organism *S. coelicolor* has been genetically engineered in a manner that positively affects antibiotic production in general (by altered RNA polymerase and ribosome) (Hosaka *et al*., 2009) and in addition alleviates the detection of antibiotic production (by inactivated active secondary metabolite gene clusters (*∆act ∆red ∆cpk ∆cda*)) (Gomez‐Escribano and Bibb, 2012). Heterologous expression of antibiotic gene clusters has been a challenge for a long time because cluster sizes can easily reach more than 100 kb, which makes them difficult to clone. However, nowadays more elaborated methods are available, as, for example, the transformation‐associated recombination cloning system, which is a method recently adopted from *Saccharomyces cerevisiae* that promotes cloning and expressing large DNA fragments in actinomycetes (Yamanaka *et al*., 2014). Besides that, the information available from gene cluster analyses can purposefully be used to manipulate pathway‐specific regulators or implement ‘refactory bricks’, for example, replacing the natural promoter with a strong artificial one, in order to directly activate the transcription of the biosynthesis gene cluster (Rutledge and Challis, 2015). An example for the activation of a silent glycopeptide cluster was described for *Amycolatopsis japonicum*. The introduction of a glycopeptide‐specific transcriptional activator resulted in the production of ristomycin, a glycopeptide (Spohn *et al*., 2014) used for the diagnosis of von Willebrand disease and Bernard–Soulier syndrome. In addition to these targeted approaches, a whole array of more pleiotropic strategies can be applied in order to activate silent gene clusters: for instance microbial co‐cultivation; addition of rare earth elements or nutrition signals, such as the cell wall metabolite N‐acetylglucosamine; or application of stress conditions (e.g. ethanol or heat stress treatment) have successfully been show to elicit silent gene cluster expression (Ochi and Hosaka, 2013; Abdelmohsen *et al*., 2015; Rutledge and Challis, 2015). Altogether, this allows to exploit the natural product producers more intensively as it has been possible in the years before and will lead to the identification of quite a number of new secondary metabolites in the near future.

### Alternative methods for drug discovery

The major disadvantage of most computational genome mining tools is that they are only able to identify biosynthetic gene clusters, which code for already known biosynthetic principles and thus the associated products may be structurally familiar compounds. However, compounds encoded by novel and unique biosynthesis mechanisms cannot be uncovered with these tools.

An alternative way for the identification of yet unknown biosynthetic pathway classes is based on the knowledge of gene regulation principles instead of occurrence and structural composition of biosynthetic enzymes. It follows the idea that global, environmental signal‐sensing regulators control the production of certain secondary metabolites. Such regulators promote or repress gene transcription by binding to specific DNA motifs upstream of their target genes in response to an environmental signal, such as nutrient starvation, oxidative stress or the presence of competitive organisms. The identification of the regulator, the determination of the specific DNA‐binding motifs and the computational screening of genome sequences for known DNA‐binding motifs of such regulators are a progression, which is defined as Identification of Natural compound Biosynthesis pathways by Exploiting Knowledge of Transcriptional regulation (INBEKT) approach (Spohn *et al*., 2016).

The application of INBEKT allowed for instance the identification of the biosynthetic gene cluster of the zincophore ethylene diamine disuccinic acid ([*S,S*]‐EDDS). [*S,S*]‐EDDS synthesis in the producer strain *A. japonicum* is known to be strictly regulated by the zinc regulator Zur. The identification of the Zur‐binding motifs in the *A. japonicum* genome finally led to the successful discovery of the [*S,S*]‐EDDS biosynthetic genes (Spohn *et al*., 2016). The [*S,S*]‐EDDS structure indicated a novel and unique biosynthesis mechanism different from the typical NRPS or NIS (NRPS‐independent siderophore synthetase) pathways known for the synthesis of chelating agents.

One of the emerging areas in microbiology is detecting specialized metabolites produced by microbial colonies and communities with MS. The newly developed imaging MS and real‐time MS allow two‐ and three‐dimensional visualization of the distribution of metabolites from microbial colonies and enable the identification of metabolites directly from microbial colonies (Yang *et al*., 2009; Fang and Dorrestein, 2014).

## Dereplication in natural product discovery

The different approaches listed in the previous chapter are leading to the synthesis of bacterial metabolites, which then can be analysed for their biological activity. However, before employing them in extensive tests, it has to be examined whether they are really new. For this dereplication, effective novel methods have been developed.

Recently, various bioinformatics approaches have been developed to organize or interpret large sets of MS/MS fragmentation data. For example, solutions such as MAGMa (MS annotation based on *in silico* generated metabolites) allow matching of multistage fragmentation data against candidate molecules substructures and were successfully applied on complex extracts (van der Hooft *et al*., 2012; Ridder *et al*., 2014; Allard *et al*., 2016). Among these new approaches, molecular networking (MN) is a particularly effective one to organize MS/MS fragmentation spectra. MN compares all MS/MS spectra in a given extract and groups them according to their similarity (Watrous *et al*., 2012; Bandeira, 2011; Liu *et al*., 2013; Fang and Dorrestein, 2014). The application of these tools leads to the identification of novel compounds by avoiding re‐isolation of known compounds.

## Further aspects of antibiotic drug development

In addition to the progress in finding new secondary metabolites, also new ideas came up and old strategies revived, which may help in the search for novel compounds or in their more effective application.

- In order to overcome the resistance problem, compounds that do not kill the pathogen but only prevent its pathogenic action may be used. This will reduce the pressure on the pathogen to acquire mutations, which will allow it to survive in the presence of the drug. Such ‘antivirulents’ may be used in combination with ‘classical’ antibiotics.
- Another strategy to reduce the occurrence of resistance is the development of new drug combinations. This has been proven to be successful in the treatment of tuberculosis and HIV, but is not a routine procedure in other medicating infections.
- Apparently, many of the most effective drugs used in human therapy have more than one target in the bacterial cell. A criterion for the future selection of drug candidates may be, therefore, the interaction with more than one target. The newly emerging research field of ‘cell biology of antibiotic action’ will deliver a deeper understanding on the mode of action of many antibiotics. This should enable the development of new approaches for the search for novel antibiotics.
- According to recent predictions Gram‐negative pathogens will constitute a major threat in future. Although Gram‐positives presently cause the majority of deaths in clinics, a greater arsenal is required to combat Gram‐negatives. A critical step which prevents the application of many compounds in Gram‐negatives is the passage across the outer membrane. Here, the combination of known antibiotics with substances, which permeabilize the outer membranes, may help. Alternatively, antibiotics can be combined with siderophore structures that promote the uptake. This ‘trojan horse’ strategy has successfully been applied in model systems.

## Challenges

**Figure 1:**
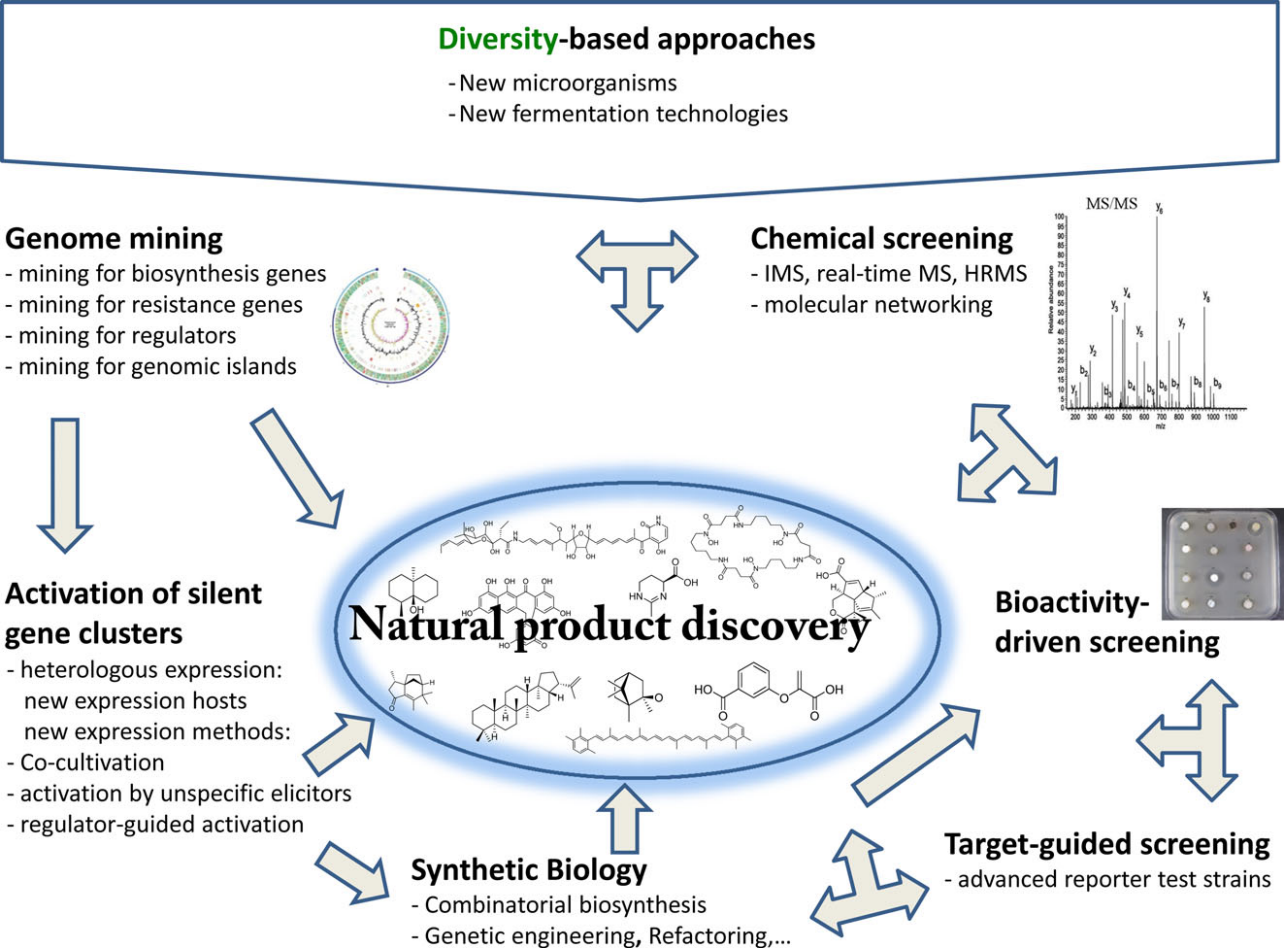
Approaches in natural product discovery aiming to identify novel antibiotics.

There is no doubt that we will need novel antibiotics in future and that natural products are most likely the best source. After a long period of steady decline in identifying novel compounds, we are now in a lucky situation: Genome mining revealed a much bigger potential to synthesize natural products than have been isolated with conventional approaches; new approaches in microbiology, in particular microbial cultivation techniques, made much more producers accessible as antibiotic producers; genetic technologies (combinatorial biosynthesis, synthetic biology, precursor‐directed biosynthesis), as well as biochemical knowledge, enable large‐scale derivatization of new and old natural compounds to optimize them in a way that they can be applied in medical applications. Presently, we have a significantly enlarged arsenal for the discovery of new antibiotics (Fig. 1). Worldwide, scientists will apply and further develop these technologies. This will undoubtedly result in the identification of novel secondary metabolites in the next years. Whereas the new tools have mainly been developed by academia, we will need cooperation between academia and industry to generate the large numbers of compounds, which are required to end up with an antibiotic that makes it to the hospital.

The described advances in the field significantly increased the probability to find something new, but many drawbacks in drug discovery have still to be overcome. This includes scientific drawbacks, but even more important economic and regulatory aspects.

The majority of antibiotics did not end up in the hospitals since severe side‐effects prevented their application. Unfortunately, no rationale is available which reliably predicts such effects from the chemical structure of new compounds.

Whenever a new antibiotic has been found, resistance developed in the pathogens, even if first evidence existed, that this would not happen. We, therefore, need better strategies to prevent resistance development such as much more sensible prescription and application of antibiotics in human medicine and an absolute strict regulation of antibiotic therapies in veterinary medicine. Since decades, big pharma concentrated on the search for broad‐spectrum antibiotics due to economic reasons (‘block busters’). In order to overcome the ‘resistance crisis’, it will be important that newly discovered antibiotics will also be further developed if they combat only a narrow spectrum of pathogens.

Fortunately, the regulatory processes to approve new antibiotics have been alleviated in recent years. They can be approved if they are ‘non‐inferior’ to already approved drugs; they do not need to be ‘superior’. In addition, special procedures are available for a more simple and quick approval in the case of limited use. This will increase the number of antibiotics which can be applied and thereby facilitate the treatment of resistant pathogens.

Taken together, the recent scientific advances in finding and characterizing new compounds which can serve as antibiotics and the progress in understanding pathogenic processes, there is hope that the pessimistic prediction on the defeat against infections does not come true.

## Conflict of interest

The authors declare no conflict of interest.
